# Factors associated with positive perceptions of sealant use on permanent molars among Brazilian dentists

**DOI:** 10.1590/1807-3107bor-2025.vol39.054

**Published:** 2025-06-02

**Authors:** Larissa Yumi ITO, Leticia Maíra WAMBIER, Ana Cláudia Rodrigues CHIBINSKI, Manoelito Ferreira SILVA, Denise Stadler WAMBIER

**Affiliations:** (a) Universidade Estadual de Ponta Grossa – UEPG, School of Dentistry, Postgraduate Program in Dentistry, Ponta Grossa, PR, Brazil.; (b) Universidade Estadual de Ponta Grossa – UEPG, Department of Dentistry, Ponta Grossa, PR, Brazil.; (c) Universidade Estadual do Sudoeste da Bahia – UESB, Department of Health I, Jequié, BA, Brazil.

**Keywords:** Pit and Fissure Sealants, Dental Caries, Perception, Dentists

## Abstract

This study aimed to analyze the factors associated with the perception among Brazilian dentists of the preventive or therapeutic use of sealants on permanent molars. A cross-sectional web-based survey was conducted with Brazilian dentists between July and October 2021 to examine their use of social media and gather data regarding sealant-related practices. A structured questionnaire was developed and applied to collect information on participants’ professional profiles, clinical indications, materials, techniques, and perceptions of pit and fissure sealants. The study outcomes were the positive perception of sealant use as: (a) a preventive measure, or (b) a therapeutic approach to carious lesions. Responses were dichotomized into positive perception (“strongly agree” or “agree”) and negative perception (“neither agree nor disagree,” “disagree” or “strongly disagree”). Independent variables included sociodemographic, educational, professional, and technical characteristics. Multiple logistic regression models were used to analyze associations (p < 0.05). A total of 2,394 dentists participated in the study, 82.5% of whom had a positive perception of sealants for prevention, and 83.1%, for therapeutic purposes. A greater likelihood of positive perception of preventive sealant use was observed among educators, those employed in public health services, dentists who “always” performed sealant application, and those who used resin sealant, glass ionomer cement, or flowable resin. Conversely, professionals working in capital cities had a lower likelihood of a positive perception. Professionals from cities with populations exceeding 500,000 inhabitants, interior areas, or metropolitan regions were less likely to have a positive perception of therapeutic sealant use. However, those who used resin sealants, glass ionomer cement, or flowable resin were more likely to perceive sealants positively. In conclusion, the positive perception of sealant use for prevention was associated with educational, professional, and technical factors, whereas the positive perception of therapeutic use was associated with professional and technical factors.

## Introduction

Despite a significant reduction in the prevalence and severity of dental caries over the past five decades, untreated caries remains widespread worldwide, and continues to rank among the most common health conditions affecting the global population. Marked inequalities persist between developed and developing countries.^1^ In Brazil, caries experience has declined over the last three decades; however, in 2010, 12-year-olds had an average of 1.67 permanent teeth affected by caries, with 61.8% of these teeth being decayed. Additionally, as age increases, there is a notable progression in the number of affected teeth, accompanied by a growing proportion of missing teeth.^2^ Tooth loss significantly impairs quality of life by affecting nutrition, speech, and aesthetics.^3^

Molars are the teeth most frequently affected by caries,^4^ primarily due to the anatomical characteristics of their occlusal surfaces. Pits and fissures in posterior teeth hinder effective hygiene, promoting biofilm accumulation. Furthermore, the enamel at the base of these pits and fissures is thinner, increasing susceptibility to carious lesion development and progression.^5,6^ Early eruption during childhood and location of molars in non-aesthetic areas often lead to an undervaluation of their care.^7^

Advancements in prevention and therapeutic techniques for carious lesions have emphasized minimal intervention approaches.^8^ Pit and fissure sealants have gained prominence due to their straightforward application technique, which preserves tooth morphology. These fluid materials act as physical barriers, preventing the penetration and retention of food debris and biofilm.^8,9^ Moreover, they inhibit mineral loss and the progression of carious lesions.^8^

A variety of materials is available for use as pit and fissure sealants, including resin-based sealants, glass ionomer sealants, flowable resins, adhesives, and bioactive materials with surface pre-reacted glass (S-PRG) particles. However, current studies^9,10^ remain inconclusive regarding the superiority of one material over another, since no specific material has been scientifically established as ideal for this purpose.

The properties required for an effective surface sealant include biocompatibility, retentive capacity, resistance to abrasion and wear, low viscosity to facilitate penetration into the pits and fissures of teeth, low solubility to oral fluids, and ease of application.^11^ Protocols for sealant application vary depending on the selected material and the characteristics of the tooth or carious lesion.^12^

The continuous evolution of materials, techniques, and indications for pit and fissure sealants may influence professional perceptions, leading to the underuse or misuse of this procedure in clinical practice.^13^ Evidence-based sealant approaches to sealant use can enhance professionals’ perceptions of its effectiveness, ultimately reducing the costs associated with treating carious lesions compared to conventional restorative techniques.^14^ However, little is known about dentists’ knowledge and perceptions regarding the use of sealants.^15-17^

Dentists’ perceptions can significantly influence decision-making regarding indications, material selection, and, consequently, patient oral health outcomes^14-17^. Given the substantial advancements in cariology and minimal intervention concepts, it is crucial to determine whether these technical advances and scientific findings are being incorporated into the daily practices of Brazilian dentists. Therefore, the objective of the present study was to analyze the factors associated with dentists’ perception of the preventive or therapeutic use of sealants on permanent molars in Brazil.

## Methods

### Study Design

This study employed a quantitative, observational, cross-sectional, and analytical approach. Data were collected through an online form completed by a sample of practicing dentists in Brazil. The study adhered to the recommendations outlined in the Strengthening the Reporting of Observational Studies in Epidemiology (STROBE) guidelines.^18^

### Ethical Considerations

The research protocol was submitted to and approved by the Research Ethics Committee of the State University of Ponta Grossa (UEPG) (CAAE: 47271321.0.0000.0105).

### Population and Sample

A sample calculation was conducted to determine the minimum number of participants required for data collection. As of May 2021, the population of dentists in Brazil was reported to be 336,249 professionals, according to the Federal Council of Dentistry. The sample size calculation assumed a heterogeneous population distribution (50%), a 95% confidence level, and a 2% margin of error, resulting in a minimum sample size of 2,384 professionals. The sample was non-probabilistic, since participants were recruited through social media.

### Eligibility Criteria

**Inclusion criteria:** Dentists actively practicing in Brazil.

**Exclusion criteria:** Participants from the pilot study and duplicate responses.

### Questionnaire

Data were collected on dentists’ knowledge regarding the indications and techniques for sealant use, their frequency of performing pit and fissure applications, and their perceptions of procedure effectiveness for preventive and therapeutic purposes. An unpublished research questionnaire was developed and administered, consisting of 63 questions addressing the research topic. The questionnaire included both closed- and open-ended questions, organized into 9 blocks: (a) professional profile: gender, type of institution where the participant graduated, primary Brazilian state of practice, years since graduation, level of qualification, place(s) of practice, and specialty(ies); (b) general use of pit and fissure sealants: frequency of application and type of pit and fissure sealant used in routine practice; (c) clinical indications for pit and fissure sealants; (d–h) materials and techniques used: specific questions on resin sealants, glass ionomer cement, flowable resin, adhesives, and resin with S-PRG particles; (i) perception of effectiveness: dentist’s perceptions of the pit and fissure sealing techniques as being preventive and therapeutic, assessed using a 5-point Likert scale (strongly agree, partially agree, neither agree nor disagree, partially disagree, strongly disagree).

A pilot study was conducted with 10 postgraduate students from the Graduate Program in Dentistry (PPGO) at UEPG (class of 2020–2021) and seven professors specializing in Pediatric Dentistry and Public Health at UEPG. Data collection was carried out by a single researcher. Participation was voluntary, and all information was kept strictly confidential to preserve participants’ anonymity. During the pilot study, any questions or uncertainties raised while completing the questionnaire were reviewed, and suggestions were analyzed by the research team to ensure alignment with the study objectives. Adjustments were incorporated into the questionnaire following three rounds of refinement. The pilot project was conducted in May and June 2021.

### Data Collection

Data collection was conducted between July and October 2021 using an online questionnaire created with Google Forms®. The questionnaire was disseminated through social networks, including Instagram, Facebook, and WhatsApp, managed by the researchers. Direct contact was made by sending the survey link via the dentist’s social media accounts. Additionally, the survey link was distributed via email to faculty members of undergraduate and graduate dentistry courses, using email addresses made publicly available on the website of their institutions.

Responses to the questionnaire were continuously monitored, and new dissemination strategies, such as active searches, were implemented to ensure proportional representation of the target population. The sample characteristics were based on data from Rizzo’s study,^19^ which analyzed sociodemographic data (distribution of professionals by gender, age group, and years since graduation), educational data, and practice location (categorized by Brazilian geographic states, macroregions, and specialty of practice).

### Variables

The study focused on two primary outcomes related to dentists’ perceptions of pit and fissure sealants on permanent molars: a) a preventive method; and b) a therapeutic method for carious lesions. The outcome was dichotomized as follows:

- Positive: Strongly agree or agree.
- Negative: Neither agree nor disagree, disagree, or strongly disagree.

### Independent Variables

The independent variables were classified into four categories:

a.**Sociodemographic characteristics**: Gender.

b.**Educational characteristics**: Type of undergraduate college, years since graduation, level of qualification, and specialty of practice.

c.**Professional characteristics**: Brazilian geographic region of practice, type and size of main municipality of practice, and type of workplace.

d.**Clinical aspects**:

- Frequency of applying pit and fissure sealants.
- Type of sealant used (invasive and/or non-invasive).
- Materials used: resin sealant, glass ionomer cement, flowable resin, adhesives, and bioactive materials containing S-PRG particles.
- Acceptable and unacceptable clinical technique steps.

The most appropriate protocols to be followed for the clinical technique steps were evaluated based on product-specific recommendations and application times. These included prophylaxis prior to the procedure, isolation, anesthesia, acid etching of the tooth surface, adhesive application, type of sealing material used, adherence to the manufacturer’s recommendations, and occlusal adjustment.

### Data Analysis

Quantitative data were tabulated using Microsoft Excel® version 16.72 and analyzed with the Statistical Package for the Social Sciences (SPSS) software for Windows, version 16.0. Closed-ended questions were evaluated quantitatively using absolute frequencies (n) and relative frequencies (%). Associations were tested using the chi-square test, with statistical significance set at p < 0.05. Following exploratory analysis, variables with p < 0.20 were included in multiple logistic regression models, which were performed with a significance level of p < 0.05. A stepwise method was employed to include and exclude variables, progressing from the most complex to the simplest models. The decision for variable retention was based on its p-value, and the Akaike Information Criterion (AIC) of the model. This analysis was conducted in the R 4.0.4 statistical environment (R Core Team, 2021).

## Results

The study received 2,405 responses. After excluding nine duplicate responses and two instances of non-concordance to participate, a total of 2,394 Brazilian dentists were included in the analysis. The majority of participants were female (64.6%), and had graduated from a public college (61.4%). A significant proportion had been practicing for 6 to 10 years since graduation (19.3%), and held a higher degree of specialization (57.9%). Most dentists worked in the private sector (69.0%). Among the specialists, the largest group specialized in Pediatric Dentistry (18.9%), followed by Orthodontics (15.3%) and Restorative Dentistry (12.9%). Participants were primarily from municipalities with populations exceeding 500,000 inhabitants (41.6%), predominantly located in the interior regions (40.9%) of the Southeast region of Brazil (37.1%) (Table 1).

**Table 1:** Sociodemographic Characteristics of Participating Dentists. Brazil, 2021.

Variable	n	%
Gender (n = 2394)		
Female	1,547	64.6
Male	847	35.4
Type of undergraduate college (n = 2,394)		
Public	1,47	61.4
Private	923	38.6
Community	1	0.0
Years since graduation (n = 2,394)		
< 1	141	5.9
1–5	432	18.0
6–10	461	19.3
11–15	422	17.6
16–20	336	14.0
21– 25	277	11.6
26–30	168	7.0
≥ 31	157	6.6
Highest degree (n = 2,394)		
Undergraduate	382	16.0
Residency	114	4.8
Specialization	1386	5.,9
Master’s	186	7.8
Doctorate	326	13.6
Specialty (n = 2,394)		
No	382	16.0
Yes	2012	84.0
Specialty (n = 3,104)*		
Pediatric dentistry	587	18.9
Orthodontics	474	15.3
Restorative dentistry	399	12.9
Public and family health	350	11.3
Dental prosthesis	301	9.7
Other specialties	993	21.0
Workplace (n = 2,833)*		
Public service	555	19.6
Private service	1954	69.0
Teaching	324	11.4
Size of main municipality of practice (in thousands of inhabitants) (n = 2 ,394)		
> 500	995	41.6
100–500	742	31.0
25–100	489	20.4
< 25	168	7.0
Type of municipality of practice (n = 2, 394)		
Capital	793	33.1
Metropolitan region (outside the capital)	622	26.0
Interior region	979	40.9
Brazilian geographical region (n = 2394)		
North	136	5.7
Northeast	639	26.7
Midwest	275	11.5
Southeast	889	37.1
South	455	19.0

*The participant could answer more than one response option.

The professionals reported that they fully or partially agreed that pit and fissure sealants on permanent molars are effective for both preventive purposes (44.8% and 37.7%, respectively) and therapeutic purposes (48.1% and 35.0%, respectively) (Figure). Table 2 presents the crude analysis of associations between dentists’ perceptions of the purposes of pit and fissure sealants in permanent molars and sociodemographic, educational, professional, and technical characteristics, conducted using the chi-square test (p < 0.05). A positive perception of sealant use in permanent molars for both preventive and therapeutic purposes in managing carious lesions was associated with several factors, such as being female (p < 0.001 and p < 0.001, respectively), having graduated within the past 0 to 10 years (p < 0.001 for both purposes), holding a higher undergraduate education level (p = 0.007 for prevention; p < 0.001 for therapy), specializing in Public Health and/or Pediatric Dentistry (p < 0.001 for both purposes), working in public service (p < 0.001 for both purposes), not working as teachers (p = 0.001 for both purposes), and practicing in municipalities located in interior regions (p = 0.023 for prevention; p = 0.022 for therapy) with populations exceeding 500,000 inhabitants (p < 0.001 for both purposes). Additionally, the aspects of consistently performing sealant application (p < 0.001 for both purposes) and employing a non-invasive technique (p < 0.001 for both purposes) were associated with a positive perception of sealant use. Furthermore, adherence to all acceptable clinical steps of the technique with glass ionomer sealants (p < 0.001) and flowable resin (p < 0.001) was exclusively associated with a positive perception of sealants for therapeutic purposes in permanent molars (Table 2).

**Figure:**
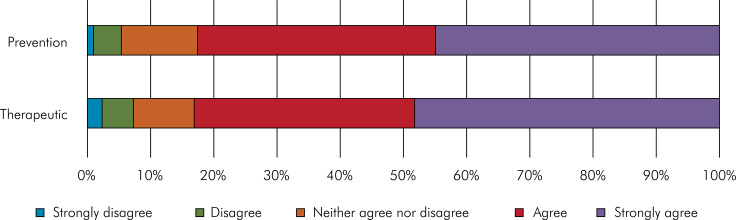
Distribution of agreement on the purpose of pit and fissure sealant use on permanent molars among dentists. Brazil, 2021.

**Table 2:** Associations between dentists’ perception of the purpose (preventive or therapeutic) of pit and fissure sealants in permanent molars and sociodemographic, educational, professional, and technical characteristics. Brazil, 2021.

Perception of pit and fissure sealants in permanent molars						
Variable	Preventive			Therapeutic		
	Positive	Negative	p-value*	Positive	Negative	p-value*
	n (%)	n (%)		n (%)	n (%)	
Gender (n = 2,394)						
Female	1,313 (84.9)	234 (15.1)	< 0.001	1,326 (85.7)	221 (14.3)	< 0.001
Male	662 (78.2)	185 (21.8)		663 (78.3)	184 (21.7)	
Type of undergraduate college (n = 2,394)						
Public	1,221 (83.1)	249 (16.9)	0.360	1,219 (82.9)	251 (17.1)	0.795
Private/Community	754 (81.6)	170 (18.4)		770 (83.3)	154 (16.7)	
Years since graduation (n = 2,394)						
0–10	896 (86.7)	138 (13.3)	< 0.001	896 (86.7)	138 (13.3)	< 0.001
11–20	612 (80.7)	146 (19.3)		631 (83.2)	127 (16.8)	
21–30	337 (75.7)	108 (24.3)		340 (76.4)	105 (23.6)	
> 31	130 (82.8)	27 (17.2)		122 (77.7)	35 (22.3)	
Highest degree (n = 2,394)						
Undergraduate	327 (85.6)	55 (14.4)	0.007	341 (89.3)	41 (10.7)	< 0.001
Residency / Specialization	1,248 (83.2)	252 (16.8)		1,256 (83.7)	244 (16.3)	
Master’s / Doctorate	400 (78.1)	112 (21.9)		392 (76.6)	120 (23.4)	
Specialty (n = 2012)						
Pediatric dentistry / Public and family health	841 (94.0)	54 (6.0)	< 0.001	843 (94.2)	52 (5.8)	< 0.001
Other specialties	838 (75.7)	279 (24.3)		849 (76.5)	268 (23.5)	
Workplace: Public service (n = 2,394)						
Yes	503 (90.6)	52 (9.4)	< 0.001	497 (89.5)	58 (10.5)	< 0.001
No	1,472 (80.0)	367 (20.0)		1,492 (81.1)	347 (18.9)	
Workplace: Private service (n = 2,394)						
Yes	1,604 (82.1)	350 (17.9)	0.266	1,625 (83.2)	329 (16.8)	0.826
No	371 (84.3)	69 (15.7)		364 (82.7)	70 (17.3)	
Workplace: Teaching (n = 2,394)						
Yes	247 (76.2)	77 (23.8)	0.001	226 (69.8)	98 (30.2)	< 0.001
No	1,728 (83.5)	342 (16.5)		1,763 (85.2)	307 (14.8)	
Size of main municipality of practice (in thousands of inhabitants) (n = 2,394)						
> 500	790 (79.4)	205 (20.6)	< 0.001	795 (79.9)	200 (20.1)	< 0.001
100–500	612 (82.5)	130 (17.5)		615 (82.9)	127 (17.1)	
< 100	573 (87.2)	84 (12.8)		579 (88.1)	78 (11.9)	
Type of municipality of practice (n = 2,394)						
Capital	634 (79.9)	159 (20.1)	0.023	638 (80.5)	155 (19.5)	0.022
Metropolitan region (outside the capital)	510 (82.0)	112 (18.0)		515 (82.8)	107 (17.2)	
Interior	831 (84.9)	148 (15.1)		836 (85.4)	143 (14.6)	
Brazilian geographic regions (n = 2,394)						
North	120 (88.2)	16 (11.8)	0.124	118 (86.8)	18 (13.2)	0.706
Northeast	529 (82.8)	110 (17.2)		532 (83.3)	107 (16.7)	
Midwest	228 (82.9)	47 (17.1)		230 (83.6)	45 (16.4)	
Southeast	714 (80.3)	175 (19.7)		738 (83.0)	151 (17.0)	
South	384 (84.4)	71 (15.6)		371 (81.5)	84 (18.5)	
Frequency of performing pit and fissure sealants (n = 2,394)						
Always	288 (97.6)	7 (2.4)	< 0.001	282 (95.6)	13 (4.4)	< 0.001
Often/Sometimes/Rarely	1,567 (89.2)	189 (10.8)		1,623 (92.4)	133 (7.6)	
Never	1,20 (35.0)	223 (65.0)		84 (24.5)	259 (75.5)	
Type of sealant used: invasive (n = 2,394)						
Yes	278 (86.6)	43 (13.4)	0.037	289 (90.0)	32 (10.0)	< 0.001
No	1,697 (81.9)	376 (18.1)		1,700 (82.0)	373 (18.0)	
Type of sealant used: non-invasive (n = 2,394)						
Yes	1,813 (90.8)	184 (9.2)	< 0.001	1,860 (93.1)	137 (6.9)	<0.001
No	162 (40.8)	235 (59.2)		129 (32.5)	268 (67.5)	
Clinical technique steps: Resin-based Sealant (n = 1,985)						
All steps acceptable	1,460 (91.0)	145 (9.0)	0.789	1,499 (93.4)	106 (6.6)	0.867
Some steps unacceptable	344 (90.5)	36 (9.5)		354 (93.2)	26 (6.8)	
Clinical technique steps: Glass Ionomer Cement (n = 1,562)						
All steps acceptable	1,326 (94.8)	73 (5.2)	0.144	1,358 (97.1)	41 (2.9)	< 0.001
Some steps unacceptable	150 (92.0)	13 (8.0)		136 (83.4)	27 (16.6)	
Clinical technique steps: flowable resin (n = 1,237)						
All steps acceptable	984 (92.7)	77 (7.3)	0.835	1,027 (96.8)	34 (3.2)	< 0.001
Some steps unacceptable	164 (93.2)	12 (6.8)		146 (83.0)	30 (17.0)	
Clinical technique steps: adhesive (n = 18)						
All steps acceptable	10 (90.9)	1 (9.1)	0.412	8 (72.7)	3 (27.3)	0.518
Some steps unacceptable	7 (100.0)	0 (0.0)		6 (85.7)	1 (14.3)	
Clinical technique steps: bioactive material with S-PRG particles (n = 41)						
All steps acceptable	9 (100.0)	0 (0.0)	0.442	9 (100.0)	-	-
Some steps unacceptable	30 (93.8)	2 (6.3)		32 (100.0)	-	-

*Chi-square test (p < 0.05)

In the final model, professionals more likely to have a positive perception of using sealants for prevention on permanent molars included educators (OR = 1 .82; 95%CI: 1.03–3.44), those working in public service (OR = 1.72; 95%CI: 1.17–2.59), and those who consistently performed the procedure (OR = 3.85; 95%CI: 1.89–9.24). Professionals residing in metropolitan areas were less likely to have a positive perception of sealants for prevention compared to those residing in capital cities (OR = 0.54; 95%CI: 0.33–0.91). Dentists who used resin-based sealants (OR = 3.20; 95%CI: 1.63–6.01), glass ionomer cement (OR = 4.20; 95%CI: 3.07–5.75), and flowable resin (OR=1.66; 95% CI: 1.21-2.27) as sealing materials demonstrated positive perceptions compared to those who did not use these materials (Table 3).

**Table 3:** Odds ratio, confidence interval, and p-value of the final model for the outcome of a positive perception of sealants on permanent molars for preventive and therapeutic purposes. Brazil, 2021.

Independent variables	OR	95%CI	p-value*
Positive perception of sealants on permanent molars for preventive purposes			
Workplace: Teaching (Yes)	1.82	1.03– 3.44	0.049
Workplace: Public service (Yes)	1.72	1.17–1.59	0.008
Type of municipality of practice: Metropolitan region (outside the capital)	0.54	0.33–0.91	0.018
Frequency of application of pit and fissure sealants (Always)	3.85	1.89–9.24	0.001
Use of glass resin sealants (Yes)	3.20	1.63–6.01	<0.001
Use of glass ionomer cement (Yes)	4.20	3.07–5.75	<0.001
Use of flowable resin (Yes)	1.66	1.21–2.27	0.002
Positive perception of sealants on permanent molars for therapeutic purposes			
Size of main municipality of practice: Over 500,000 inhabitants	0.54	0.30–0.96	0.034
Type of municipality of practice: Interior region	0.39	0.19–0.81	0.011
Type of municipality of practice: Metropolitan region (outside the capital)	0.48	0.27–0,85	0.011
Use of glass resin sealants (Yes)	3.51	1.77–6.59	< 0.001
Use of glass ionomer cement (Yes)	3.74	2.63–5.33	< 0.001
Use of flowable resin (Yes)	1.76	1.24–2.51	0.002

*Multiple logistic regression models (p < 0.05).

In the final model, professionals working in municipalities with populations exceeding 500,000 inhabitants were less likely to have a positive perception of sealants for therapeutic purposes in permanent molars (OR = 0.54; 95%CI: 0.30–0.96). Similarly, those working in inner-city areas (OR = 0.39; 95%CI: 0.19–0.81) and metropolitan regions (OR = 0.48; 95%CI: 0.27–0.85) exhibited lower odds of having positive perceptions. Conversely, dentists who used resin-based sealants (OR = 3.51; 95%CI: 1.77–6.59), glass ionomer cement (OR = 3.74; 95%CI: 2.63–5.33), and flowable resin (OR = 1.76; 95%CI: 1.24 - 2.51) as sealing materials were more likely to have positive perceptions compared to those who did not use these materials (Table 3).

## Discussion

The majority of dentists in Brazil demonstrated a positive perception of the effectiveness of pit and fissure sealants on permanent molars as a preventive and/or therapeutic method, which is consistent with findings from other international studies conducted in England,^15^ Sana’a (Yemen),^16^ and Bangalore (India).^17^ This observation reinforces that most professionals in Brazil are informed about contemporary techniques, and adopt practices guided by modern concepts that align with the philosophy of minimal intervention. Such alignment reflects progress in integrating evidence-based approaches and advancing scientific knowledge in dentistry.

However, a portion of dentists reported never performing sealants in their clinical practice. This reluctance may stem from skepticism about the effectiveness of the technique, insufficient knowledge due to outdated education or limited access to updated resources, and/or clinical specialization in areas where there is little indication for sealant use. Additional factors, such as the absence of necessary materials in the workplace, or difficulties in justifying the cost of the procedure to patients and caregivers, may further contribute to this behavior. Reliance on flawed clinical experience, rather than scientific evidence, may also lead some professionals to associate sealants with lower-quality treatment, despite the substantial evidence supporting their effectiveness.^13,17,20,21^

The higher percentage of dentists who agree on the preventive effectiveness of sealants may be attributed to advancements in cariology and improvements in material quality. Preventive effectiveness refers to sealants applied on surfaces without carious lesions but with vulnerability or risk of dental caries, in contrast to therapeutic effectiveness, which involves their use in incipient carious lesions or micro-cavitated lesions.

Dental sealants were introduced in the 1960s solely for the prevention of dental caries.^9^ However, alongside the evolution of cariology, new materials have been developed that increasingly enable the technique to be used for treating incipient carious lesions, with considerable advancements in application protocols.^8^ In this context, scientific evidence consistently supports the use of sealants in carious lesions located in the enamel or outer third of dentin (up to 3 mm), including micro-cavitated lesions in permanent teeth.^9,22^

Given the proven effectiveness of sealants for both preventing caries and halting the carious process, combined with their simplicity and low cost compared to restorative treatments,^23^ it is anticipated that more professionals will adopt minimally invasive procedures in their clinical practices. This expectation applies regardless of the workplace, whether public or private, or the dentist’s specialty, provided the patient presents with indications for this procedure. To achieve this, knowledge of caries diagnosis, including risk and activity assessment, is essential. These elements are critical for evaluating individual patients, thus ensuring intervention is performed at the optimal moment, and avoiding both undertreatment and overtreatment.^13^

This highlights the need for broader dissemination of guidelines and protocols for sealant use through continuing education courses and updates to dental school curricula. Incorporating the latest recommendations from the American Dental Association (ADA) into educational programs will better prepare professionals to perform sealant applications effectively in their clinical practice, irrespective of their specialty. To support this, protocols must be clearer and more direct, enabling dentists to build confidence through access to accurate and up-to-date information about sealant application.^13^

Regarding associated factors, teaching professionals were more likely to have a positive perception of sealant use as a preventive method for carious lesions. This is likely due to their involvement in academic and scientific environments, where there is a continuous exchange of information and new knowledge. These professionals are engaged in an ongoing cycle of continuing education, thus ensuring that recent theories and advancements are incorporated into their teaching.^24^ In this context, the pursuit of evidence-based practice is integral,^21^ particularly since the study does not distinguish whether the educators specialized in Cariology.

Dentists working in the public healthcare system also exhibited a higher prevalence of positive perceptions regarding sealant use as a preventive method. This finding suggests that the National Oral Health Policy (Política Nacional de Saúde Bucal in Portuguese) has been effective in achieving its objectives. Designed to guide and structure the oral healthcare network, this policy introduced new perspectives and strategies for organizing dental care, shifting the focus toward prevention and health promotion.^25^ Furthermore, in 2004, the National Permanent Health Education Program was established to provide continuous education and training for public health service workers. This initiative has contributed to workforce qualification, positively impacting both individual and collective healthcare.^26^ As a result, professionals working in the public sector may be more up to date and better equipped to perform sealant applications, thus influencing their more favorable perception of the technique.

Another key finding was that professionals with a positive perception of sealants were also those who most frequently performed sealant applications in their clinical practice. This underscores the importance of professional training, bearing in mind that greater knowledge is directly associated with the ability to accurately diagnose and correctly indicate sealant use in permanent molars. Additionally, increased familiarity with clinical steps and materials enhances the likelihood of successful caries prevention and therapeutic outcomes.^10^

Regarding the factors associated with the positive perception of sealant use as a therapeutic method, dentists practicing in municipalities with populations exceeding 500,000 inhabitants, in inner-city areas, and in metropolitan regions were less likely to have a positive perception. Similarly, dentists practicing in metropolitan regions were less likely to have a positive perception of sealants for prevention compared to those practicing in the capital cities. Although the literature does not provide evidence to support these findings, future national and international studies should explore whether this pattern is unique to Brazil, or is observed in other countries as well. One hypothesis is that professionals in smaller municipalities or metropolitan regions may encounter a different patient profile, such as individuals with poorer socioeconomic conditions. These patients may seek care at a later stage or have less consistent professional follow-up, which could hinder the timely implementation of minimally invasive procedures. Additionally, higher patient flow in capital cities, whether in public or private services, may prompt professionals to prioritize quick and effective procedures.^27^ Another possibility is that professionals working in major urban centers have greater access to continuing education opportunities.^28^

Professionals who use resin-based sealants, glass ionomer cement, and flowable resin tend to have more positive perceptions of sealants for both preventive and therapeutic applications. This finding suggests that professionals who trust in the effectiveness of the technique, irrespective of the type of sealing material used, are more likely to incorporate it into their practice. Additionally, it raises the hypothesis that frequent use, low cost, and versatility of these materials in other restorative procedures—such as glass ionomer cement and flowable resin—may contribute to their greater availability in clinical settings, thus facilitating their use for sealant application.

Among the main limitations of this study are: (a) the use of a new questionnaire that did not undergo all validation steps, (b) the non-probabilistic sampling method, and (c) selection bias. However, it is important to emphasize that the objective was to describe general aspects of the study subject rather than to develop or validate a research instrument. Additionally, although the study employed a calculated sample size and met the minimum required number of participants, it was conducted as a web survey with data collection via social media. Consequently, the results cannot be inferred as representative of Brazilian dentists. Nonetheless, it is noteworthy that the sample obtained in this research reflects the national profile of dental professionals^19^, since the participant demographics were tracked throughout data collection.

The greater participation of women has been observed in other web surveys, and, in this case, aligns with the actual distribution of professionals in Brazil.^19,27^ Furthermore, higher participation of younger professionals is expected in studies of this nature, given their greater familiarity with and time spent on social media.^29,30^ In Brazil, where the number of dental schools has expanded exponentially over the past two decades,^27,28^ this trend was reflected in the higher participation of professionals with less time since graduation (up to 15 years). There was also a greater proportion of professionals from the Southeast region and from municipalities with populations exceeding 500,000 inhabitants, which is consistent with areas that have a higher concentration of dental schools and professionals.^27,28^ Although this expansion has been more pronounced in private colleges,^27^ the majority of respondents reported attending public colleges. One possible explanation is that students from public institutions are more engaged in and accustomed to research environments, whereas research involvement is not a mandatory component of private college curricula. Additionally, while the public sector is the largest employer of dental professionals in Brazil,^28^ a higher proportion of participants in this study reported working in the private sector. This finding is consistent with other studies in which participants were recruited through social media platforms such as Instagram.^29,30^ Finally, the researchers identified the need to explore the barriers faced by professionals who do not perform sealant application, an aspect that should be addressed in future studies.

## Conclusion

The majority of dentists practicing in Brazil demonstrated a positive perception of pit and fissure sealant use on permanent molars. Factors associated with a positive perception of sealants for preventive purposes were linked to educational, professional, and technical characteristics. In contrast, factors associated with a positive perception of sealants for therapeutic purposes were related to professional and technical characteristics.
